# Role of radiation therapy in primary breast diffuse large B‐cell lymphoma in the Rituximab era: a SEER database analysis

**DOI:** 10.1002/cam4.1457

**Published:** 2018-04-06

**Authors:** Pan‐pan Liu, Ke‐feng Wang, Jie‐tian Jin, Xi‐wen Bi, Peng Sun, Yu Wang, Hang Yang, Zhi‐ming Li, Wen‐qi Jiang, Yi Xia

**Affiliations:** ^1^ Department of Medical Oncology Sun Yat‐sen University Cancer Center 651 Dong Feng East Road Guangzhou Guangdong 510060 China; ^2^ State Key Laboratory of Oncology in South China Guangzhou Guangdong 510060 China; ^3^ Collaborative Innovation Center for Cancer Medicine Guangzhou Guangdong 510060 China; ^4^ Department of Thoracic Surgery The Sun Yat‐sen Memorial Hospital of Sun Yat‐sen University Guangzhou 510120 China

**Keywords:** Primary breast diffuse large B‐cell lymphoma, radiation, SEER analysis, survival

## Abstract

Primary breast diffuse large B‐cell lymphoma (PB‐DLBCL) is an uncommon extranodal non‐Hodgkin's lymphoma (NHL), which was traditionally treated with anthracycline‐containing regimens followed by consolidative radiation therapy (RT) to add therapeutic benefits. The introduction of anti‐CD20 antibody rituximab for the treatment of B‐cell NHLs has significantly improved the clinical outcome of these malignant diseases. It is unclear, however, whether consolidative RT could still add therapeutic benefits for PB‐DLBCL patients treated with rituximab. To answer this important question, we used the Surveillance, Epidemiology, and End Results (SEER) database to evaluate the impact of RT on the clinical outcomes of PB‐DLBCL patients in the rituximab era. Information on patient age, year of diagnosis, stage, race, laterality, and RT status for PB‐DLBCL patients diagnosed between 2001 and 2014 were extracted. Kaplan–Meier survival curves were plotted, and log‐rank test was used to compare the potential survival difference. Multivariate analysis using Cox proportional hazards model was employed to determine the impact of RT and other factors such as age, race, tumor laterality, stage, and year of diagnosis on survival. Among the 386 patients identified, the median follow‐up time was 45 months (range, 0–167 months); the median age was 64 years (range, 19–93 years); 33.9% of the patients were younger than 60 years of age; 69.9% of the patients were stage I; 79.0% were white; 51.8% received RT. The 5‐year OS and cause‐specific survival (CSS) for the whole cohort were 72.3% and 82.5%, respectively. The 5‐year OS was significantly superior for patients who received RT compared to those who did not receive RT (78.1% vs. 66.0%, *P *=* *0.031). In multivariable analysis, RT remained significantly associated with improved OS (*P *=* *0.026). In summary, our study suggests that RT still adds significant therapeutic benefits for patients with PB‐DLCBL in the rituximab era.

## Introduction

Primary breast lymphoma (PBL) was first defined by Wiseman and Liao in 1972 as a malignant lymphoma limited to the breast or the breast and ipsilateral axillary lymph nodes, but without concurrent disseminated disease 1. This is a relatively rare but significant disease with a reported incidence of 0.5% of all breast malignancies and 2% of extranodal non‐Hodgkin's lymphomas (NHLs), with DLBCL being the most common histologic subtype 1, 2, 3. PB‐DLBCL mostly occurs in women with a median age of 62–64 years at diagnosis in Western countries and 45–53 years in East Asia 2, 3, 4, 5, 6, 7, 8. PB‐DLBCL shows poorer outcome compared with nodal DLBCL in the prerituximab era. This type of extranodal lymphoma has a high risk of contralateral breast and central nervous system (CNS) relapse.

Due largely in part to the low incidence, there is a lack of prospective studies to guide the optimal treatment. It seems that radical resection should be avoided, as retrospective studies have demonstrated that mastectomy offered no benefit over nonmastectomy treatment in terms of the overall survival (OS) 3, 5. Combined treatment consisting of chemotherapy and RT seems to be the treatment of choice in the prerituximab era. For instance, Alives conducted a controlled study and found that combination therapy using six cycles of CHOP plus RT of 30 Gy could induce a higher rate of complete remission (CR) and a lower rate of relapse compared with RT alone or chemotherapy alone 9. In agreement with this result, a study by the International Extranodal Lymphoma Study Group (IELSG‐15) also recommended consolidative RT followed by chemotherapy based on the observation that RT was associated with better OS and a trend to improve progression‐free survival (PFS) and cause‐specific survival (CSS) and reduced the risk of ipsilateral progression 3. Further studies also suggested that PB‐DLBCL would benefit from such combined therapy 2.

The anti‐CD20 antibody rituximab was tested in the management of B‐cell NHLs in late 1990s and has since been confirmed to improve OS by 10–30% for most of B‐cell NHLs. Several studies have assessed the value of rituximab in PB‐DLBCL 7, 8, 10, 11, 12, 13. The use of rituximab in chemotherapy tends to prolong PB‐DLBCL patients' PFS and OS 13, suggesting that rituximab might change the biological behaviors of this disease. As the successful application of rituximab has been shown to improve the outcome of DLBCL, it is important to evaluate whether RT could still add therapeutic benefits for PB‐DLBCL patients treated with rituximab or whether RT might only increase the risk of toxic side effect without additional therapeutic benefits. In this study, we used the Surveillance, Epidemiology, and End Results (SEER) database to evaluate the impact of RT on the clinical outcome of PB‐DLBCL patients in the rituximab era.

## Methods

### Data source

The source of data for our study was from 18 SEER databases of the National Cancer Institute in the United States. SEER is a program that collects and publishes cancer incidence, treatment, and survival data from population‐based cancer registries, representing approximately 28% of the US population. The 18 registries in SEER‐18 include approximately 25% of white population, 26% of black population, 38% of Hispanic population, 44% of American Indians and Alaska (A/PI) population, 50% of Asians, and 67% of Hawaiian/Pacific Islanders. The 18 SEER registries including Atlanta, Detroit, Greater California, Greater Georgia, Hawaii, Iowa, Kentucky, Los Angeles, New Mexico, New Jersey, Rural Georgia, states of Connecticut, San Francisco‐Oakland, Seattle‐Puget Sound, San Jose‐Monterey, the Alaska Native Tumor Registry, Louisiana, and Utah were used in this study.

### Study cohort

The SEER database uses the third edition of the International Classification of Disease for Oncology (ICD‐O‐3) to classify cancer histology and tomography. Patients with PB‐DLBCL in this study were identified using ICD‐O‐3 codes for histology (9680, diffuse large B‐cell lymphoma [DLBCL], NOS; 9684, malignant lymphoma, large B, diffuse, immunoblastic; 9688, T‐cell histiocyte‐rich large B‐cell lymphoma) and for anatomic location in the breast (ICD‐O‐3 topography code: C50).

**Figure 1:**
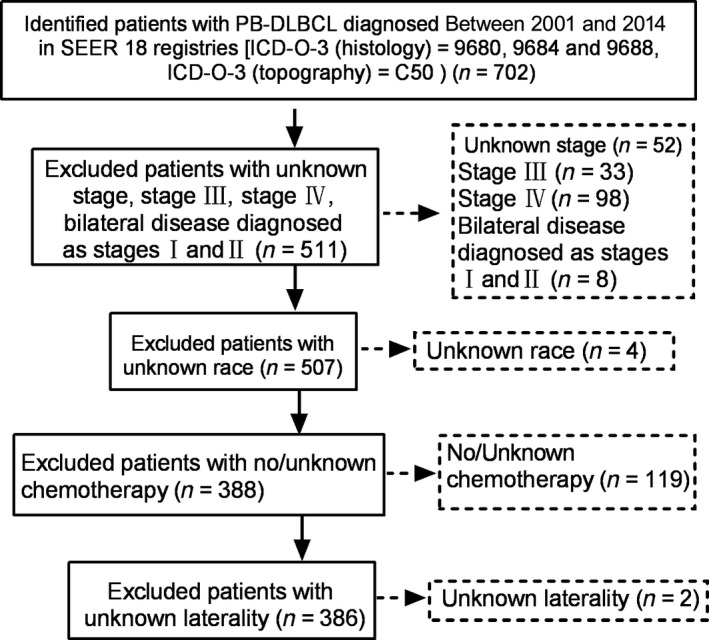
Patient selection flowchart.

For this study, we included patients with PB‐DLBCL diagnosed between 2001 and 2014, a period expected to reflect frequent applications of rituximab in the treatment of lymphomas. Patients with unknown stage, no exact information on disease laterality, stage III, stage IV and bilateral disease diagnosed as stages I and II, with unknown race/ethnicity, younger than 18 years, or received no chemotherapy were excluded (Fig. 1). The variables of age, race, laterality, stage, RT, and year of diagnosis were subjected to both univariate and multivariate analyses to assess their prognostic value on survival.

### Statistical analysis

Statistical analysis was performed using SEERstat 8.3.4 and Statistical Package for the Social Sciences (SPSS) version 20.0 software (IBM Corporation, Armonk, NY). Kaplan–Meier survival curves were plotted, and log‐rank test was used to compare the survival difference. Multivariate analysis using Cox proportional hazards model was used to determine the impact of RT, race, age, sex, stage, and year of diagnosis on survival. A *P* value of <0.05 was considered statistically significant.

## Results

**Figure 2:**
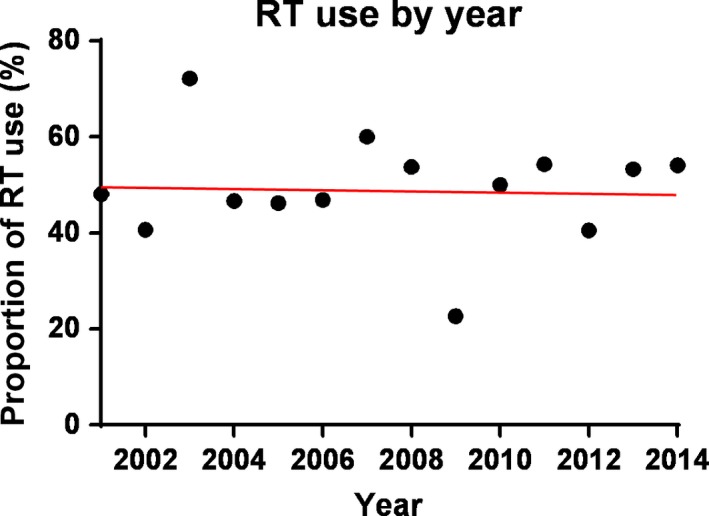
Constant proportion of RT use in PB‐DLBCL by year.

From 2001 to 2014, a total of 386 patients with stage I and II PB‐DLBCL were reported to the SEER registries. The median follow‐up time was 45 months (range 0–167 months). The data showed that this disease often occurred in older patients, with a median age of 64 years (range 19–93 years). Of these patients, 79.0% were of white race and 69.9% presented with stage I disease. The disease involvement in the right breast was as often as in the left breast. Approximately half of the patients (51.8%) received RT. The use of RT remained relatively constant (48–54%) over time (Fig. 2). The basic characteristics between patients received RT and those without RT were compared as shown in Table 1. There were no statistical differences between RT group and non‐RT group in terms of age, race, tumor laterality, disease stages, and year of diagnosis.

**Table 1:** Demographic and clinical characteristics of primary breast DLBCL patients with or without radiation therapy (RT) from 2001 to 2014

Variable	Total, *n*	RT, *n* (%)	No RT, *n* (%)	*P*
		200 (51.8)	186 (48.2)	
Age, years				
<60	131	68 (34.0)	63 (33.9)	0.532
≥60	255	132 (66.0)	123 (66.1)	
Race				
White	305	157 (78.5)	148 (79.6)	0.448
Nonwhite	81	43 (21.5)	38 (20.4)	
Tumor laterality				
Left	188	93 (46.5)	95 (51.1)	0.213
Right	198	107 (53.5)	91 (48.9)	
Stage				
I	270	151 (75.5)	119 (64.0)	0.009
II	116	49 (24.5)	67 (36.0)	
Year				
2001–2005	117	60 (30.0)	57 (30.6)	0.489
2006–2014	269	140 (70.0)	129 (69.4)	

### Clinical outcomes

**Figure 3:**
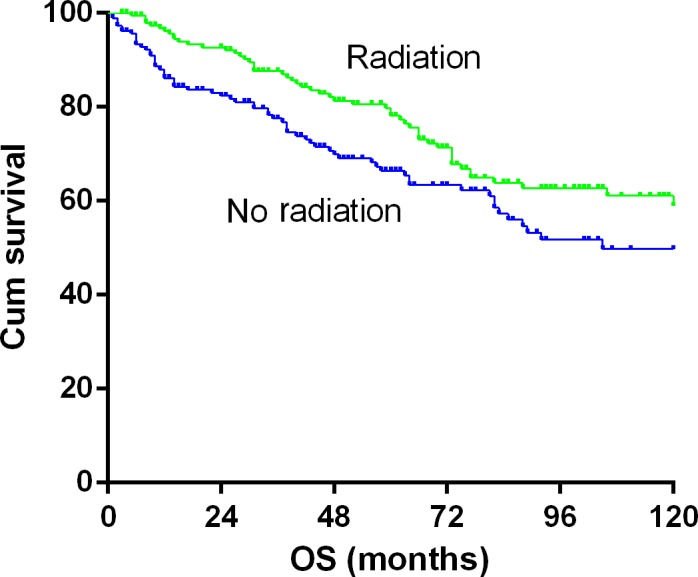
Kaplan–Meier survival curves of PB‐DLCBL patients treated with and without RT.

The 5‐year OS and CSS rates for the whole cohort were 72.3% and 82.5%, respectively (Fig. S1). Kaplan–Meier analysis showed that there was no significant difference in OS between the groups of right and left laterality, stages I and II, and year of diagnosis of the 2001–2005 and 2006–2014 groups. OS significantly decreased in patients with age ≥60 years and in the white race patients (Fig. S2). When Kaplan–Meier survival curves were plotted for the RT and non‐RT groups, the data revealed that RT significantly improved OS (Fig. 3, *P* = 0.031). Univariate analysis showed that RT was associated with better OS [RT versus non‐RT: HR = 0.680 (0.478–0.969)] (Table 2).

**Table 2:** Univariate and multivariate analyses of prognostic factors in PB‐DLBCL patients

Variable	5‐year OS (%)	Univariate analysis		Multivariate analysis	
		Hazard ratio (95% CI)	*P*	Hazard ratio (95% CI)	*P*
Age, years					
<60	84.0	1	<0.001	1	<0.001
≥60	67.5	3.224 (2.024–35.136)		3.173 (1.983–5.075)	
Race					
White	69.5	1	0.008	1	0.068
Nonwhite	83.1	0.532 (0.322–0.880)		0.618 (0.373–1.026)	
Laterality					
Right	70.9	1	0.387	1	0.713
Left	73.7	0.856 (0.602–1.218)		0.936 (0.657–1.222)	
Stage					
I	76.3	1	0.104		0.119
II	63.0	1.373 (0.944–1.997)		1.353 (0.925–1.980)	
Radiation					
No RT	66.0	1	0.017		0.026
RT	78.1	0.680 (0.478–0.969)		0.668 (0.469–0.953)	
Year					
2001–2005	70.9	1	0.376		0.101
2006–2014	74.7	0.844 (0.581–1.227)		0.731 (0.502–1.063)	

In multivariate Cox regression analysis (Table 2), RT was significantly and independently associated with improved OS (HR 0.668, 95% CI 0.469–0.953). This was consistent with the results of univariate analysis. Patients younger than 60 years of age were also independently associated with better OS (Table 2).

## Discussion

Using the traditional Wiseman and Liao's definition 1, our study mainly focused on lymphoma patients with the disease localized to the breasts and the regional lymph nodes. We did not include systemic diseases with breast involvement, as in such cases the primary disease sites are unclear. Thus, patients with Ann Arbor stage III and IV were excluded. The rare incident of PB‐DLBCL makes it difficult to conduct large randomized studies to determine the optimal treatment. Although prospective clinical trials were initiated with a purpose to assess the impact of rituximab in the de novo PB‐DLBCL with or without RT, such attempts failed as the studies were stopped early due to slow enrollment (https://clinicaltrials.gov/ct2/show/NCT01279772; https://clinicaltrials.gov/ct2/show/NCT01144754). Results from retrospective studies indicate that rituximab tends to prolong OS of PB‐DLBCL. It is unknown whether the use of rituximab in PB‐DLBCL might make it unnecessary to add RT, which could induce additional toxic side effect. In this study, we conducted a large population‐based analysis and found that adding RT was associated with the improvement of overall survival in the rituximab era. Thus, consolidative RT might still be recommended for PB‐DLBCL patients treated with rituximab.

Consistent with the published results, we also found PB‐DLCBL mostly occurred in elderly patients, with a median age of 64 years. The majority of the patients presented with stage I. Tumor laterality distributed equally between left and right breasts (48.7% and 51.3%, respectively). Patients older than 60 years showed inferior outcome. The patients of white race seem to exhibit worse outcome associated with older age.

Treatment strategies for PB‐DLBCL have evolved during the past decades. Surgical intervention should be avoided, as surgery seems to be associated with high cause‐specific mortality and inferior outcome. Combined modality approaches such as chemotherapy in combination with RT produce superior outcomes for PB‐DLBCL. Chemotherapy is the backbone of lymphoma treatment, and regimen consisting of anthracycline was associated with improved outcome. RT in combination with chemotherapy might further consolidate chemotherapy response. A large retrospective IELSG‐15 study underscores the importance of consolidative RT in the management of PB‐DLBCL 3. It was found that RT plus anthracycline‐containing chemotherapy was associated with improved OS compared to RT or chemotherapy alone. A controlled study by Aviles et al. 9 also confirmed that RT followed by six cycles of CHOP regimen was associated with significantly improved outcomes. Multiple studies have demonstrated the benefit of adding rituximab to chemotherapy regimen in DLBCL of different sites 14, 15, 16, 17. Aviles et al. 9, 10 conducted a prospective study and found that addition of rituximab to the intensive chemotherapy regimen (CEOP‐14) offered no benefit in terms of complete response rate and OS. Several retrospective studies reported that addition of rituximab showed a tendency to prolong the progression‐free survival (PFS) and OS of PB‐DLBCL. A small‐scale study showed that rituximab could significantly improve PB‐DLBCL patients' PFS and OS, with the 5‐year PFS and OS of 82.4% for 6 patients who received R‐CHOP and 67.5% for 15 patients who received CHOP (*P *=* *0.038) 8. These results should be interpreted with caution due to the small number of patients in this study. In the prerituximab era, PB‐DLBCL patients were considered to have inferior clinical outcome and high rates of local and CNS relapse compared with nodal DLBCL 3, 18. Yhim et al. 13 found that the difference in outcome disappeared when rituximab was integrated into systemic chemotherapy. They treated PB‐DLBCL and nodal DLBCL patients with R‐CHOP and showed a similar OS between the two groups (3‐year OS rate, 82.2% vs. 90.7%, respectively; *P *=* *0.345). The reason for such similar clinical outcome in the rituximab era might be that most PB‐DLBCL exhibited a nongerminal center B‐cell‐like (non‐GCB) phenotype, and multiple studies have confirmed that rituximab might overcome the adverse prognostic impact of the non‐GCB phenotype 16, 19, 20. Although there is a lack of prospective study to confirm the efficacy of rituximab in PB‐DLBCL, it is still recommended to add rituximab to traditional chemotherapy regimen for PB‐DLBCL treatment due to the high effectiveness of rituximab in eliminating B lymphoma cells and reduce the CNS relapse risk.

In line with the published results, our analysis of the SEER database also revealed the change in treatment modalities for PB‐DLBCL over the past decades (Fig. S3). The use of surgery has declined to under 10%, and RT increased to about 50%. Although RT is commonly used in the management of this disease, it should be noted that not all patients received RT. From 2001 to 2014, the use of RT remained constant at about 50% of PB‐DLBCL patients (Fig. 2). When giving treatment recommendations, many oncologists often consider lung and cardiovascular toxicity associated with RT. Potential risk of secondary malignancies is another concern. Nevertheless, it seems appropriate to recommend RT for PB‐DLBCL patients with stage I disease, as these patients can benefit from consolidative RT. It is possible that involved site radiotherapy (ISRT) to PB‐DLBCL patients without prophylactic RT to the uninvolved regional lymph nodes or contralateral breast could reduce the risk of radiation‐related toxicities to contralateral breast, lung, and thyroid gland.

Our study selected the year 2000 as a cutoff time, when rituximab began to be widely used in the clinic. The patients diagnosed with PB‐DLBCL from 2001 to 2014 were included in this analysis. Survival analysis of the SEER database shows favorable outcomes, with a 5‐year OS of 72.3% for the whole cohort, which is comparable with the previously published results. A summary of several large studies on PB‐DLBCL is given in Table 3. The 5‐year OS varied from 50% to 78.1%. The 5‐year OS of 78.1% for PB‐DLBCL patients in the RT group represents the best results reported.

**Table 3:** Treatment and outcome of analysis of primary breast DLBCL

Study	Time period	Age, years	DLBCL (% of series), *n*	Chemotherapy regimens	Rituximab (%)	RT (%)	OS (%)
SEER	2001–2014	67	386 (100%)	Unknown		49.2	72.3, 66.0 (no RT), 78.1 (RT) (5 year)
Ryan 3	1980–2003	64	204 (100)	70% anthracycline based	0%	64	63 (5 year)
Yhim 7	1994–2009	48	49^a^ (100)	97% anthracycline based	61.8%	30.9	74.3 (5 year)
Validire 18	1986–2004	62	38 (83)	80% anthracycline based	10%	71	OS 61^a^ (5 year)
Zhao 8	1977–2007	54	28 (90)	CHOP 74%	% not stated	65	71 (5 year)^a^
Guo 21	1991–2006	47	37 (82.2)	CHOP‐like 79%	14%	49	50 (5 year)^a^
Aviles 9	1988–1995	58	96	RT v CHOP v combined			50, 50, 76 (10 year)
Aviles 10	1999–2002	56.7	32 (100)	R‐CEOP Q14	100%	100	63 (3 year)

a Data include non‐DLBCL histologies.

When we analyze the impact of RT on clinical outcome, we observed a significant improvement in OS in patients received RT (HR 0.680, 95% CI 0.478–0.969, *P *=* *0.017). The 5‐year OS was 78.1% and 66.0% for patients treated with or without RT, respectively. Interestingly, factors such as age, race, tumor laterality and stage did not affect the difference between RT and no RT groups (Table 1), thus decreasing the potential bias affecting prognosis derived from disease characteristics. The survival rate at 5 years observed in the RT group is the best‐reported outcome for patients with PB‐DLBCL (Table 3). The 12.1% improvement of 5‐year survival in the RT group was significant. We also for the first time evaluated the influence of tumor laterality of breast on survival and showed similar disease involvement of left and right breasts.

Previous study suggested that stage II might be a bad prognostic factor for PB‐DLBCL. Our study showed that the 5‐year OS for stage II was seemingly inferior to stage I, but such difference was not statistically significant. We also found that age older than 60 years was correlated with the poor outcome. Interestingly, patients of white race exhibited relatively worse OS compared to the nonwhite patients. A possible explanation is that the median age at diagnosis is generally older in white patients, as older age was associated with worse OS.

It should be noted that this population‐based study based on SEER database has some limitations. First, information of specific treatment regimen and the time course of treatment are lacking. This makes it difficult to determine what proportions of PB‐DLBCL patients actually received rituximab and anthracycline‐containing chemotherapy. However, it is reasonable to assume that most patients received anthracycline‐based chemotherapy, as our study excluded the patients with no or unknown chemotherapy. Another limitation is that the volume and dosing of RT for those patients were unavailable in SEER database. Also, the detailed pathological information was not available in SEER database. As DLBCL consists of many subtypes such as double‐ or triple‐hit large B‐cell lymphoma, the aggressive property of this kind of lymphoma could significantly affect the clinical outcome and thus could complicate data interpretation.

To our knowledge, this SEER‐based analysis is the first large retrospective study to evaluate the role of RT in patients with PB‐DLBCL in the rituximab era. Our study revealed that RT was associated with better OS, which was confirmed in both univariate and multivariate analyses. From 2001 to 2014, only about half of PB‐DLBCL patients received RT. As RT could significantly improve treatment outcome regardless of age, stage, tumor laterality, and race, it seems that the other half of the PB‐DLBCL patients could benefit from adding RT to the treatment protocols. Before the available of prospective randomized study to confirm the impact of rituximab and RT on PB‐DLBCL, it seems beneficial to give consolidative RT in combination with immunochemotherapy in PB‐DLBCL patients.

## Conflict of Interest

No conflict of interest to be declared.

## Supporting information


**Figure S1.** Survival of primary breast diffuse large B‐cell lymphoma.

**Figure S2.** Kaplan Meier curves for overall survival in patients with PB‐DLCBL. A: by race; B. by age.

**Figure S3.** Treatment modalities for PB‐DLBCL patients over time.

## References

[1] Wiseman, C. , and K. T. Liao . 1972 Primary lymphoma of the breast. Cancer 29:1705–1712. 4555557 10.1002/1097-0142(197206)29:6<1705::aid-cncr2820290640>3.0.co;2-i

[2] Aviv, A. , T. Tadmor , and A. Polliack . 2013 Primary diffuse large B‐cell lymphoma of the breast: looking at pathogenesis, clinical issues and therapeutic options. Ann. Oncol. 24:2236–2244. 23712546 10.1093/annonc/mdt192

[3] Ryan, G. , G. Martinelli , M. Kuper‐Hommel , R. Tsang , G. Pruneri , K. Yuen , et al. 2008 Primary diffuse large B‐cell lymphoma of the breast: prognostic factors and outcomes of a study by the International Extranodal Lymphoma Study Group. Ann. Oncol. 19:233–241. 17932394 10.1093/annonc/mdm471

[4] Cheah, C. Y. , B. A. Campbell , and J. F. Seymour . 2014 Primary breast lymphoma. Cancer Treat. Rev. 40:900–908. 24953564 10.1016/j.ctrv.2014.05.010

[5] Jennings, W. C. , R. S. Baker , S. S. Murray , C. A. Howard , D. E. Parker , L. F. Peabody , et al. 2007 Primary breast lymphoma: the role of mastectomy and the importance of lymph node status. Ann. Surg. 245:784–789. 17457172 10.1097/01.sla.0000254418.90192.59 PMC1877073

[6] Jung, S. P. , M. Kim , K. M. Han , J. H. Kim , J. S. Kim , S. J. Nam , et al. 2013 Primary breast lymphoma: a single institution's experience. J. Korean Surg. Soc. 84:267–272. 23646311 10.4174/jkss.2013.84.5.267 PMC3641365

[7] Yhim, H. Y. , H. J. Kang , Y. H. Choi , S. J. Kim , W. S. Kim , Y. S. Chae , et al. 2010 Clinical outcomes and prognostic factors in patients with breast diffuse large B cell lymphoma; Consortium for Improving Survival of Lymphoma (CISL) study. BMC Cancer 10:321. 20569446 10.1186/1471-2407-10-321 PMC2927999

[8] Zhao, S. , Q. Y. Zhang , W. J. Ma , M. H. Zhang , W. Z. Sun , H. B. Li , et al. 2011 Analysis of 31 cases of primary breast lymphoma: the effect of nodal involvement and microvascular density. Clin. Lymphoma Myeloma Leuk. 11:33–37. 21454188 10.3816/CLML.2011.n.004

[9] Avilés, A. , S. Delgado , M. J. Nambo , N. Neri , E. Murillo , and S. Cleto . 2005 Primary breast lymphoma: results of a controlled clinical trial. Oncology 69:256–260. 16166814 10.1159/000088333

[10] Avilés, A. , C. Castañeda , N. Neri , S. Cleto , and M. J. Nambo . 2007 Rituximab and dose dense chemotherapy in primary breast lymphoma. Haematologica 92:1147–1148. 17650450 10.3324/haematol.10892

[11] Schmitz, N. , S. Zeynalova , B. Glass , U. Kaiser , E. Cavallin‐Stahl , M. Wolf , et al. 2012 CNS disease in younger patients with aggressive B‐cell lymphoma: an analysis of patients treated on the Mabthera International Trial and trials of the German High‐Grade Non‐Hodgkin Lymphoma Study Group. Ann. Oncol. 23:1267–1273. 21989328 10.1093/annonc/mdr440

[12] Villa, D. , J. M. Connors , T. N. Shenkier , R. D. Gascoyne , L. H. Sehn , and K. J. Savage . 2010 Incidence and risk factors for central nervous system relapse in patients with diffuse large B‐cell lymphoma: the impact of the addition of rituximab to CHOP chemotherapy. Ann. Oncol. 21:1046–1052. 19861575 10.1093/annonc/mdp432

[13] Yhim, H. Y. , J. S. Kim , H. J. Kang , S. J. Kim , W. S. Kim , C. W. Choi , et al. 2012 Matched‐pair analysis comparing the outcomes of primary breast and nodal diffuse large B‐cell lymphoma in patients treated with rituximab plus chemotherapy. Int. J. Cancer 131:235–243. 21823120 10.1002/ijc.26352

[14] Coiffier, B. , E. Lepage , J. Briere , R. Herbrecht , H. Tilly , R. Bouabdallah , et al. 2002 CHOP chemotherapy plus rituximab compared with CHOP alone in elderly patients with diffuse large‐B‐cell lymphoma. N. Engl. J. Med. 346:235–242. 11807147 10.1056/NEJMoa011795

[15] Dunleavy, K. , S. Pittaluga , L. S. Maeda , R. Advani , C. C. Chen , J. Hessler , et al. 2013 Dose‐adjusted EPOCH‐rituximab therapy in primary mediastinal B‐cell lymphoma. N. Engl. J. Med. 368:1408–1416. 23574119 10.1056/NEJMoa1214561 PMC4568999

[16] Saito, B. , E. Shiozawa , T. Usui , H. Nakashima , T. Maeda , N. Hattori , et al. 2007 Rituximab with chemotherapy improves survival of non‐germinal center type untreated diffuse large B‐cell lymphoma. Leukemia 21:2563–2566. 17597802 10.1038/sj.leu.2404844

[17] Sehn, L. H. , J. Donaldson , M. Chhanabhai , C. Fitzgerald , K. Gill , R. Klasa , et al. 2005 Introduction of combined CHOP plus rituximab therapy dramatically improved outcome of diffuse large B‐cell lymphoma in British Columbia. J. Clin. Oncol. 23:5027–5033. 15955905 10.1200/JCO.2005.09.137

[18] Validire, P. , M. Capovilla , B. Asselain , Y. Kirova , R. Goudefroye , C. Plancher , et al. 2009 Primary breast non‐Hodgkin's lymphoma: a large single center study of initial characteristics, natural history, and prognostic factors. Am. J. Hematol. 84:133–139. 19199367 10.1002/ajh.21353

[19] Nyman, H. , M. Adde , M. L. Karjalainen‐Lindsberg , M. Taskinen , M. Berglund , R. M. Amini , et al. 2007 Prognostic impact of immunohistochemically defined germinal center phenotype in diffuse large B‐cell lymphoma patients treated with immunochemotherapy. Blood 109:4930–4935. 17299093 10.1182/blood-2006-09-047068

[20] Winter, J. N. , E. A. Weller , S. J. Horning , M. Krajewska , D. Variakojis , T. M. Habermann , et al. 2006 Prognostic significance of Bcl‐6 protein expression in DLBCL treated with CHOP or R‐CHOP: a prospective correlative study. Blood 107:4207–4213. 16449523 10.1182/blood-2005-10-4222 PMC1895783

[21] Guo, H. Y. , X. M. Zhao , J. Li , and X. C. Hu . 2008 Primary non‐Hodgkin's lymphoma of the breast: eight‐year follow‐up experience. Int. J. Hematol. 87:491–497. 18414980 10.1007/s12185-008-0085-4

