# Supplementary figures and images for: Role of radiation therapy in primary breast diffuse large B‐cell lymphoma in the Rituximab era: a SEER database analysis
Source: Cancer Med. 2018 Apr 6;7(5):1845–51. doi: 10.1002/cam4.1457 (PMC5943465; doi:10.1002/cam4.1457)

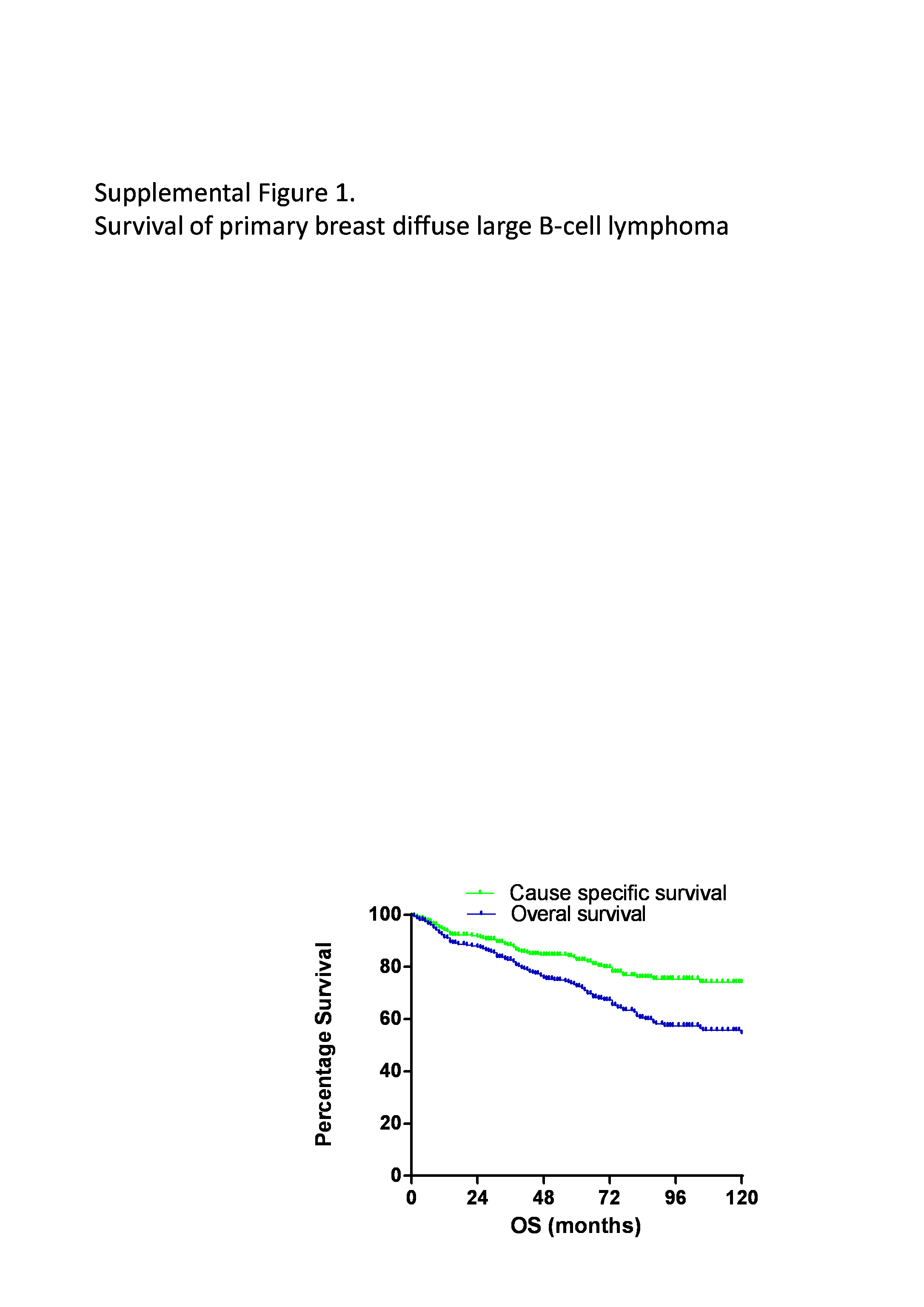

Supplement: Supplementary file 1 — Figure S1. Survival of primary breast diffuse large B‐cell lymphoma. [file CAM4-7-1845-s001.TIF]

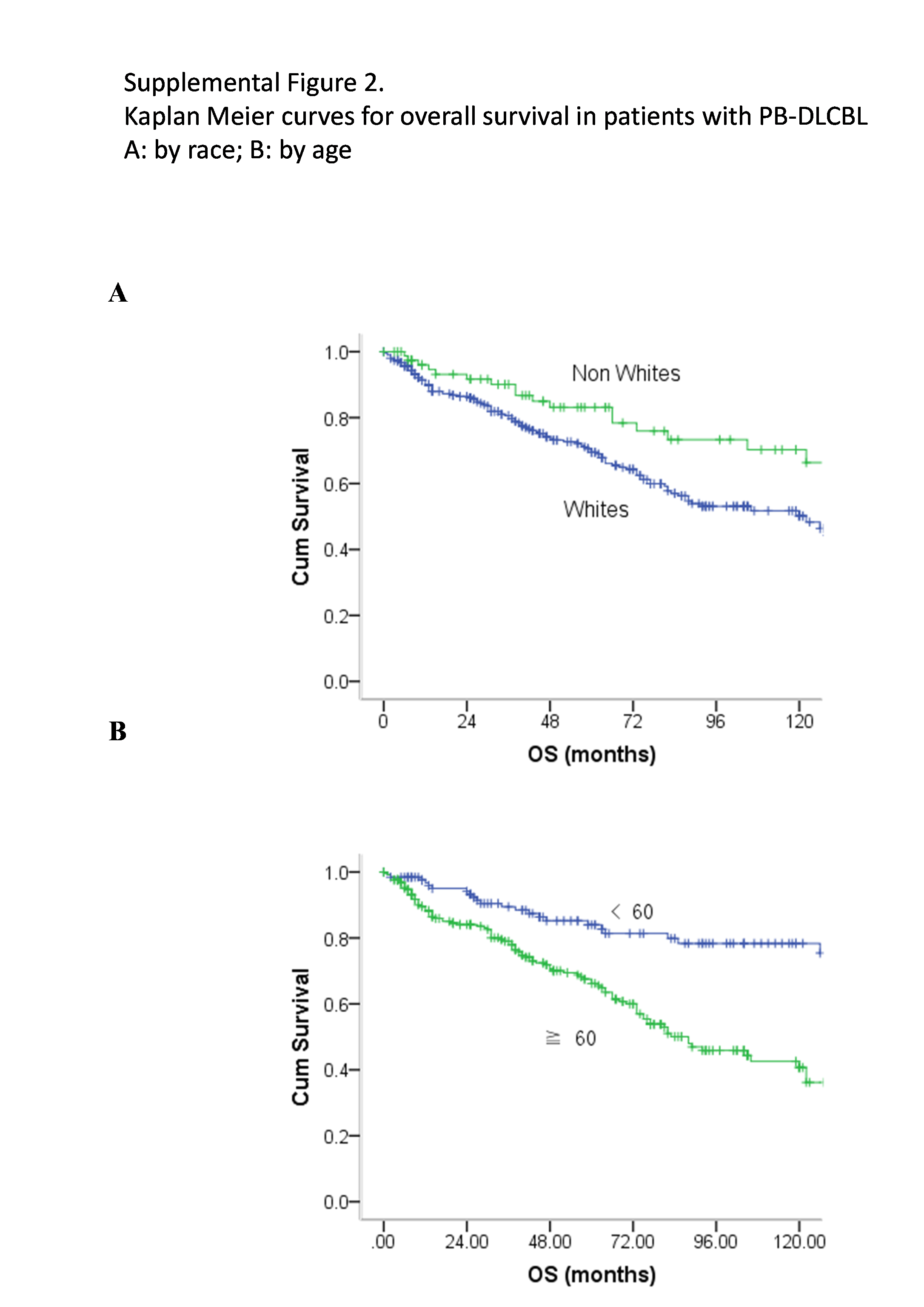

Supplement: Supplementary file 2 — Figure S2. Kaplan Meier curves for overall survival in patients with PB‐DLCBL. A: by race; B. by age. [file CAM4-7-1845-s002.TIF]

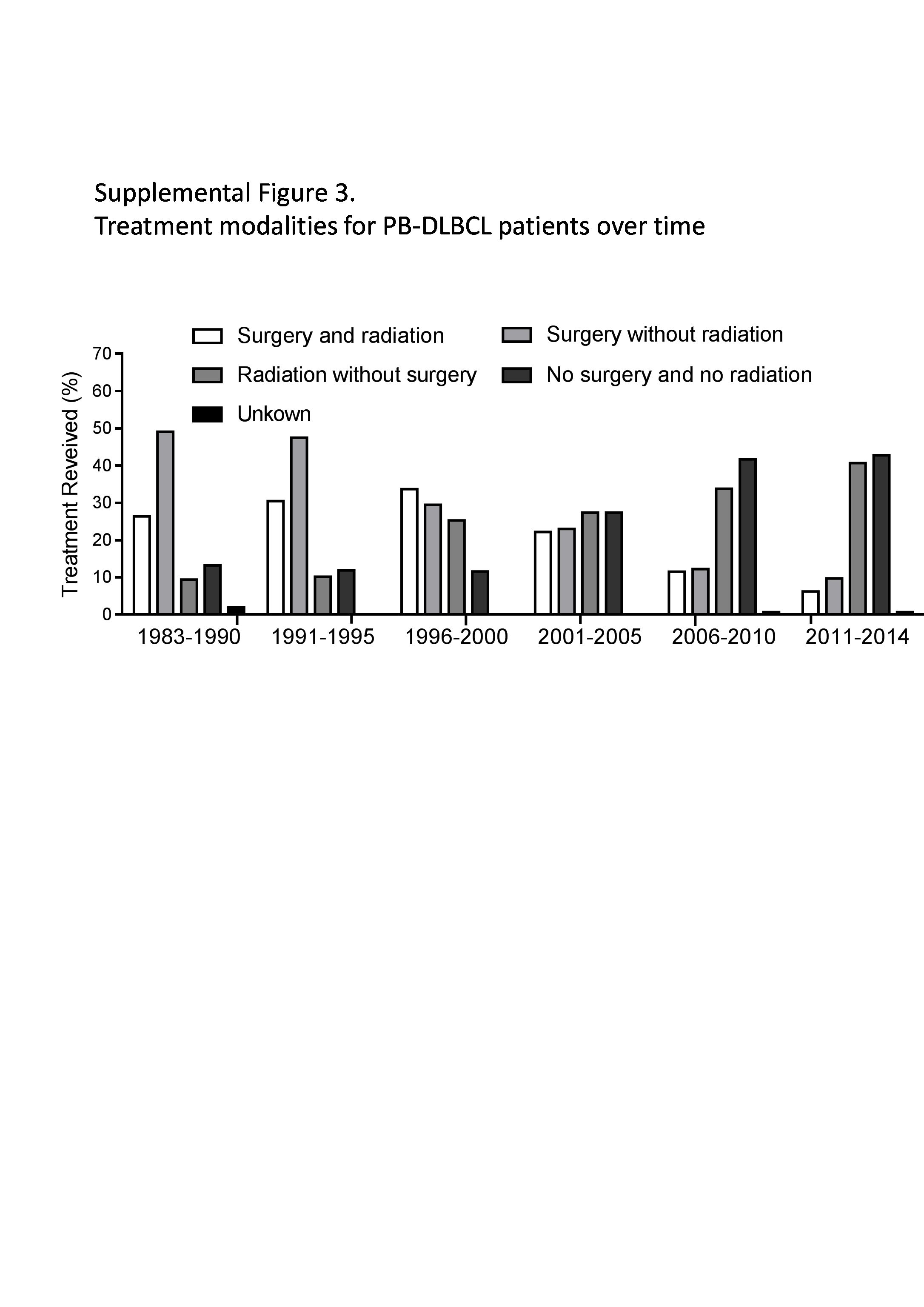

Supplement: Supplementary file 3 — Figure S3. Treatment modalities for PB‐DLBCL patients over time. [file CAM4-7-1845-s003.TIF]
